# Enhancing Learning Systems in Using Patient Experience Data: An Exploratory Mixed‐Method Study in Two Italian Regions

**DOI:** 10.1002/hpm.3912

**Published:** 2025-02-25

**Authors:** Elisa Peruzzo, Milena Vainieri, Sabina De Rosis

**Affiliations:** ^1^ Management and Health Laboratory Institute of Management Sant'Anna School of Advanced Studies Pisa Italy

**Keywords:** knowledge management, learning system, mixed method, patient experience data, quality improvement

## Abstract

In the quest for healthcare systems enhancement, the improvement of patient experience plays a central role. The challenge lies in converting patient‐reported experience data into actionable knowledge for quality improvement. This study aims to investigate the use of patient‐reported data as knowledge base for actions and to identify and map actions derived from the use of patient‐experience data within two Italian regional healthcare systems. Patient Experience Data are systematically collected in both systems, providing real‐time updates accessible by professionals and managers through web‐based reporting systems and including a collaborative network among practitioners. A sequential exploratory mixed‐method study was carried out in several qualitative and quantitative phases. In the first phase, a qualitative method was conducted to discuss the actionability of patient‐reported data and to design a tool for collecting the improvement actions based on these data. In the second phase, a quali‐quantitative survey was performed to explore the professionals' use of patient‐reported information and the types of actions implemented. Finally, a workshop was held to discuss, interpret and validate the results. The initial workshop identified key dimensions for improvement initiatives. After design and distribution of survey, a total of 189 responses was collected, respectively 96 from Region A and 93 from Region B. Both regions ensured widespread use of patient‐reported data (89%). The establishment of a collaborative network seemed to reduce the learning curve in using patient‐reported data and fostered a culture of using patient feedback effectively. The results reveal a difference between the two regions, with a more extensive patient‐reported data use in Region A, attributed to its systematic joining the PREMs Observatory, prior experiences with patient‐feedback collection and use, and patient‐experience indicators integrated into the performance evaluation system. Regarding practices of data use, four themes emerged, namely, internal actions addressed to hospital staff (35.9%), external actions addressed to users (18.6%), comfort and hospitality aspects (34.7%) and review of processes and procedures (10.8%). The study highlights the importance of effectively using patient‐reported data to achieve organisational goals, by combining different managerial strategies. It demonstrates how professionals use such data for improvement actions and underscores the significance of various forms of knowledge dissemination and sharing. It advocates for fostering a culture of continuous learning and improvement within and across healthcare organisations.

**Summary**

- Tracking the patients' experience is necessary for improving healthcare services in their perspective.
- Addressing barriers to patient experience data use enhances actions for the improvement of healthcare service quality.
- The dissemination of knowledge based on patient‐reported inputs promotes a culture of patient‐oriented improvement.
- The combination of managerial mechanisms of control and knowledge dissemination increases the adoption of patient input more than the second approach alone.

## 1 Introduction

In recent decades, the imperative to improve healthcare systems has become more pronounced relative to the past [1]. Central to this effort, measuring and monitoring healthcare services' user experience is fundamental to identifying crucial aspects, positive aspects and areas for improvement [2]. In order to improve the quality of services, from the patient perspective also, it's essential to treat patient feedback as a crucial source of information, to share it as key knowledge within the healthcare organisations and to translate input into output.

The knowledge‐based theory of the organisation asserts that organisations derive their structure and success from their ability to effectively manage knowledge [3]. Mechanisms for integrating knowledge within organisations are crucial for success, as is the recognition that knowledge is the most strategically significant resource [4].

In the healthcare sector, several mechanisms have been implemented, on the one hand, to create and disseminate information throughout the whole organisation and, on the other hand, to incentivise their knowledge, use and create impact. It has been demonstrated that the dissemination of patient‐reported data has the potential to positively affect the professionals' behaviours [5]. Brännback (1999) underlined that organisational success depends on user‐oriented approach, which must be disseminated across the organisation as common knowledge [6].

Common knowledge encompasses elements of information that every member of the organisation should possess. It plays a crucial role in facilitating the sharing of knowledge that may not be universally known [4]. Secondly, levers for increasing the uptake of these knowledge can include both technological supports to the access to data [7, 8, 9], and performance evaluation systems, monetary incentives related to specific goals, benchmarking approaches to push on the reputational levels [10]. These latter are typical mechanisms of performance management, fall within the realm of management control [11] and have been widely adopted in different care settings, such as the hospital one [12, 13]. This includes also the uptake of patients' perspective into the performance evaluation and management programs [14, 15, 16, 17, 18].

Individuals, rather than organisations as a whole, are considered the primary source of knowledge creation and utilisation, presenting challenges in coordinating and integrating diverse knowledge [4]. The learning organisation approach promotes self‐directed learning processes with a key role of single individuals at the ‘micro’ level of the organisation [19]. In other words, achieving truly open learning systems means that the individuals engaged in the learning process drive the transformation of the organisation, rather than the organisation ‘using’ them for shaping the organisation.

The aim of this study is twofold: first, to investigate the utilisation of patient‐reported information as knowledge‐base for actions and, second, to explore the types of actions implemented. The study was multi‐centric, involving healthcare organisations in two contexts, where different methods for knowledge dissemination and management have been introduced.

### 1.1 Learning From Patient Experience

In healthcare setting, nowadays the patients' voice is mainly collected using Patient‐Reported Experience Measures (PREMs) as a tool that allows to capture the standard dimensions of patient experience [7, 20]. This tool enables the investigation of the quality of care and service from the patients' perspective.

Nevertheless, despite many healthcare organisations and professionals prioritise the measurement of the patient experience, the real challenge lies in converting this data into actionable knowledge for enhancing quality [21, 22]. Moreover, the research on the use of patient‐reported data mainly stresses the presence of several organisational, professional and practical barriers [23, 24, 25, 26, 27]. Organisational barriers include characteristics of the organisation and the services provided, organisational culture, available resources and interactions among stakeholders [28, 29, 30]. Professional obstacles encompass individual‐related, work‐related and team‐related barriers [31, 32]. Practical barriers can be related to data collection and data reporting [7, 10, 33]. According to Bastemeijer et al., addressing these barriers may positively influence the effectiveness of quality improvement initiatives that target patient experiences [27].

Patients' feedback can have the potential to drive improvement actions and initiatives at various levels, from individual patient (micro), to organisational strategies (meso) and broader healthcare system and policies (macro) [17]. In literature, there are studies that report initiatives, activities, action plans and projects of using patients' feedback for diverse purposes [26, 34]. Firstly, patients' feedback is instrumental in improving hospital comfort and hospitality aspects on the wards. This includes improving access, waiting time, food service, cleanliness, lighting, parking facilities, noise reduction, privacy preservation [35, 36]. Secondly, patient‐reported data can contribute to enhance accountability and transparency. The public disclosure of information is important for ‘making visible’ the results of public participation into survey for evaluating and possibly improving services [37]. Thirdly, patient feedback can serve for ameliorating the quality of communication between professionals and patients/caregivers. This entails paying attention to dignity, courtesy and kindness [38]. Lastly, patient‐reported data can inform initiatives targeted at hospital staff, such as training courses programed to promote the cultural and professional growth, increase staff motivation, enhance staff education and cultivate a favourable work environment [39].

Approaches based both on the knowledge diffusion and on control‐related models have been presented as practices to promote the knowledge‐based improvement of organisations and individuals.

On the one hand, previous studies have underlined the importance of systematic and continuous collection of PREMs for enhancing the possibility of data use [7]. The systematic collection and return of patient feedback has been designed to promptly provide healthcare managers and professionals with actionable insights to manage critical care aspects and to operationalise the improvements [8]. Here, professionals play a crucial role in the adoption and success of systematic patient feedback collection systems [40]. Practices like these latter, which are based on professionals' commitment and trust, enable experimentation, autonomy, involvement and knowledge sharing [41].

On the other hand, recent studies showed that specific values‐based approaches, including control actions, can bring about relevant behavioural changes in the healthcare professionals [41]. In fact, patient‐report measures have progressively integrated as indicators of hospital performance into the Performance Evaluation Systems (PES) [16]. Patient‐indicators have been utilised for benchmarking and accreditation goals, which are powerful levers for managing people and change behaviours [31, 32].

Some academics suggest embracing a commitment‐based work structure to increase professionals' skills and capabilities, such as routines and processes, which foster commitment and trust more effectively than traditional control‐oriented management practices [42, 43].

Considering these premises, the aims of this research are to investigate, within a real‐world setting, the data use and map initiatives and practices of using patient experience data to enhance health services and patients' experiences. This study seeks to understand if different managerial practices of knowledge dissemination and/or control contribute to the general utilisation or to specific utilisation of this information.

## 2 Methods

### 2.1 Setting

The Italian National Health Service follows the Beveridge model, functioning as a public health system providing universal coverage for essential health services through general taxation. Since the 1990s, a strong decentralisation policy has been adopted in Italy and gradually transferring authority from the central state to its 20 regions. Consequently, each Italian region adopts its own healthcare governance model [44].

The context of this study is the Italian PREMs Observatory on hospitalisation, which began in 2018 and was joined by two Italian regional healthcare systems at the time of this research. For each region, healthcare managers and professionals were involved from one teaching hospital (TH), three Local Health Authorities (LHAs) and the hospitals managed by the LHAs (Supporting Information S2: Table 1). A total of 8 healthcare organisations and 48 hospitals were included in the study. To ensure anonymity, the names of the regions and healthcare organisations are anonymised.

Both regions gradually joined the Observatory in 2018, but Region A initially more systematically than Region B. The Regions participating in the Observatory collect patient data continuously, enabling a substantial dataset. From 2018 to 2023, in Region A more than 436,000 patients were enroled and almost 126,000 patients have answered the PREMs survey with a response rate of 29%. In Region B more than 177,600 patients were enroled and more than 62,300 patients have answered the PREMs survey with a response rate of 35%. Professionals, including clinicians and nurses, are engaged in the PREMs Observatory from the phase of patient information and enrolment into the survey, to the uptake of patient‐information into their actions.

The PREMs questionnaire is composed by items of experience and satisfaction with hospitalisation service, in addition to questions on socio‐demographic characteristics (e.g., sex, age, level of education). The complete questionnaire is available in De Rosis et al. [7]. The collection and use of data are supported in both regions by digital platform that provides real‐time patient data to managers and professionals at all the levels of the system: healthcare organisations, hospitals and wards [7]. In the web platform, raw data are provided in a descriptive way, using graph bar charts and tables. Regarding open‐ended questions, the platform displays all feedback reported by patients. Quantitative and qualitative data are shown by date and setting. Tables and comments can be downloaded. Practitioners can access, consult and additionally analyse patient‐reported data. Access is limited to credentialled users (Supporting Information S1: Figure 1).

The PREMs Observatory not only involves the collection and reporting of patient feedback but also includes at least two collaborative workshops per year with representatives from each participating healthcare organisation. During these workshops, results are discussed and interpreted, and participants share practices for increasing professionals' and patients' involvement and for using the data. Additionally, these two regional healthcare systems have joined the Italian interregional network that shares a common system for the evaluation and management of healthcare performance. The network meets around three times a year and aims to foster a regional and organisational culture about understanding and using data, also those reported by patients. This expands the discourse and ideas surrounding patient‐reported data interpretation and utilisation to involve other practitioners than those directly involved into the PREMs Observatory.

One of the Regions joining the PREMs Observatory has integrated patient indicators from the PREMs Observatory into the PES from its inception [16, 40]. Patient indicators include both monitoring indicators related to patient participation in the survey and indicators of experience related to performance of certain aspects of care during hospitalisation. The indicators are presented in benchmarking among healthcare organisations, hospital and settings (i.e., medical and surgical), and with evaluation colour bands. Practitioners can access to patient‐reported indicators using another web platform, which is annually updated (Supporting Information S1: Figure 2).

### 2.2 Data Collection

A sequential exploratory mixed‐method study was carried out, involving a convenience sample, namely, managers and professionals from healthcare organisations participating to the PREMs Observatory. A sequential exploratory design is a multi‐phase mixed study in which the researcher qualitatively explores the intended subject before constructing the quantitative study [45]. Since this study ultimately aims to collect and map the initiatives based on patients' feedback, greater emphasis is placed on the second phase, which holds more significance than the first. The use of both qualitative and quantitative methods (such as workshops and questionnaire) was chosen to provide a comprehensive and in‐depth understanding of the phenomenon under study [46]. This mixed‐methods approach allowed us to compare different perspectives on the topic, which is not extensively studied in literature, particularly using empirical methods.

In our study, we conducted an initial workshop, a survey and a final workshop. The workshops were facilitated by a moderator to explore attitudes, feelings and ideas on specific topics. Sessions lasted around 1.5–2 h, with a focus on participants' shared knowledge. The workshops lasted until new inputs from participants confirmed what already emerged rather than adding anything new. The moderator, a trusted researcher acting as a neutral third party, facilitated interaction by allowing participants to discuss and compare experiences and perspectives. This has helped reveal the reasoning behind their opinions and to gather insights and data. During the two workshops, two researchers took notes. While the workshops were not recorded, they were documented through reports based on the fieldnotes taken by researchers. Reports were shared with all participants for integration and final agreement.

The first phase consists of a workshop held in July 2022, in which the referents and coordinators of activities around the PREMs Observatory from the organisations joining the same initiative were invited. The main topic was to discuss the actionability of patient‐reported data. The moderator presented the current state of PREMs Observatory in Italy and encouraged participants to discuss their experiences and opportunities of patient‐reported data use in the day‐by‐day practice. Areas of applications were explored, as well as barriers and facilitators. During the workshop, participants have emphasised the need to develop a tool to systematically collect and document improvement actions based on patient‐reported data.

The discussion during the workshop informed the design of the subsequent survey. Before the distribution, the final version of the questionnaire was revised and face‐validated by three practitioners from the two healthcare regional systems. This step was aimed at checking item relevance with respect to the informative needs, representativeness of key topics, understandability and clarity of questions. The questionnaire has been slightly modified based on the feedback received to better align with the research objectives and ensure actionable results.

The questionnaire was targeted at professionals from organisations participating in the PREMs Observatory. Specifically, it was directed to professionals who have access to patient‐reported data through the online data return platforms, described above, and have at least one the following characteristics: a managerial role, competences to interpret and analyse data, possibility to implement improvements actions.

The survey consisted of both closed‐ended and open‐ended questions, allowing for both qualitative and quantitative analysis. It was composed by two sections: one directed to professionals who indicated never having used the patient‐reported data and another to professionals who reported using the data. The first group was asked about the barriers to patient‐information use, namely, unfamiliarity with patient experience measures, inexperience and lack of training to understand and analyse patient‐reported data, insufficient time and resources, inadequate tools for data collection and reporting; lack of support and motivation from management, presence of other priorities. They were also asked about the potential use in case of absence of obstacles (i.e., for solving a problem or for valuing a positive aspect). The second group was asked whether the action moved from an aspect that was criticised by patients (i.e., a negative aspect), or favourably reported by patients (i.e., a positive aspect). They were asked to describe in a narrative way the action, its objective (e.g., for valuing professionals, training, introducing a new service, evaluating external service, assessing organisational changes, reviewing internal procedures), the group of people targeted by the action, and its potential impact and eventual related evaluation mechanisms (i.e., measures, indicators, tools). These open‐ended questions were included to get as full a view as possible of using patient‐reported data, because closed‐ended survey questions by design limit the scope of what a respondent can include in his/her answers. Professionals who have used patient data several times to implement improvement actions had the opportunity to fill the survey several times: each compilation of questionnaire corresponds to a specific initiative.

In the second phase of this study, data were collected from November 2022 to February 2023 via an online web‐based platform (i.e., LimeSurvey). The results of this phase were returned, discussed and validated by regional, organisational and hospital managers and professionals during a second workshop held in June 2023. The representatives and coordinators responsible for activities related to the PREMs Observatory were invited. The workshop was aimed at sharing and validating the results about practices of patient‐reported data use, encouraging participants to consider further improvements.

### 2.3 Data Analysis

Data collected during the first workshop were analysed using thematic analysis to identify recurring themes regarding the use of patient‐reported data. In the second phase, since the questionnaire included both closed‐ and open‐ended questions, descriptive statistics were performed for quantitative data and thematic technique were used for qualitative data.

The thematic analysis aims to identify, analyse and report patterns (themes) within data that can potentially provide a rich, detailed and complex understanding of the data [47]. Data analysis was guided by the steps for conducting thematic analysis outlined by Braun and Clark (2006), namely: familiarisation with data, generating initial codes, searching for themes, reviewing themes and defining and naming themes [47]. During the first step, no coding of any type was used. In this phase, two authors independently read and become familiar with the data. In the other phases of analysis process, the responses were revisited and were assigned initial codes, which were grounded in the data. These codes facilized the identification and the naming of the key themes (i.e., Areas for improvement). Lastly, comparisons of frequency of codes were used to identify which categories of codes are most prevalent. The coding process was facilitated using the qualitative data analysis software programme NVivo 10.0. The study was reported according to the Consolidated Criteria for Reporting Qualitative Research (COREQ) [48].

## 3 Results

### 3.1 Results of the First Phase

Nineteen persons participated to the first workshop, that was held online. Specifically, 5 researchers, 10 for Region A and 4 for Region B. All participants from the regions have a managerial role within their organisation and are engaged in the implementation and monitoring of the PREMs survey.

The first workshop produced several insights on current experience and future opportunities of patient‐reported data use. The need for a more detailed mapping of actions informed by patient‐reported data emerged. Specifically, professionals asked for a tool to capture key information about initiatives, including its objective, target audience, timeline and expected impact of the action. They suggested to involve all professionals with credentials for accessing patient‐reported data from the PREMs Observatory, in order to gather initiative at all the level of the organisation: micro (i.e., the single ward), meso (i.e., the department or hospital), macro (i.e., the healthcare organisation). A structured collection of actions was proposed, also for allowing a future sharing and dissemination of practices among professionals.

Data are collected and analysed to inform the second phase.

### 3.2 Results of the Second Phase

The survey, developed after the workshop, was sent to 404 professionals of Region A. For privacy reasons, in the Region B, the invitation was sent by an internal regional organisation. A total of 189 responses were collected, respectively 96 from Region A (23.8% response rate) and 93 from Region B (Table 1). Considering that the users of Region B who had access to the data return platform were 545, we can estimate a response rate of 17.1%.

**TABLE 1:** Responses collected by Italian regions and healthcare organisations.

Region	Organisation	Respondents	
		Frequency	Percentage
Region A	LHA1	26	13.8
	LHA2	21	11.1
	LHA3	21	11.1
	TH1	28	14.8
Total region A		96	50.8
Region B	LHA4	56	29.6
	LHA5	6	3.2
	LHA6	24	12.7
	TH2	7	3.7
Total region B		93	49.2
Total of respondents		189	100

Abbreviations: LHA = local health authority, TH = teaching hospital.

Among the respondents, more than 11% (*n* = 22) declared they had never used patient‐reported feedback, despite they joined the PREMs Observatory. A difference emerged between the two Regions: respectively, 7.3% (*n* = 7) in Region A and 16.1% (*n* = 15) in Region B, showing a greater underutilisation of these data. The primary barrier to patient‐reported data use cited by professionals was the unfamiliarity and inexperience with patient experience measures (Table 2). For this group of participants, the potential utility of PREMs stays in limiting or eliminating perceived critical aspects of the hospitalisation experience more than for emphasising positive aspects, in particular for practitioners in Region B. Respondents not using this data would potentially focus their future use of patient‐data on improving informational support and involvement of patients and caregivers.

**TABLE 2:** Barriers to patient‐reported data use reported by professionals.

Barriers to the use of data		Region A		Region B		Total	
Category	Sub‐category	Frequency	Percentage	Frequency	Percentage	Frequency	Percentage
Capacity to PREMs use	Unfamiliarity with patient experience measurement systems	3	23.1	4	18.2	7	20
	Inexperience and lack of training to understand and analyse results	4	30.8	4	18.2	8	22.9
	Total	7	53.9	8	36.4	15	42.9
Lack of resources	Insufficient time and resources	3	23.1	4	18.2	7	20
	Inadequate tools for data collection and reporting	0	0	2	9.1	2	5.7
	Total	3	23.1	6	27.3	9	25.7
Top management commitment	Lack of support and motivation from management	1	7.7	0	0	1	2.9
	Presence of other priorities	2	15.4	3	13.6	5	14.3
	Total	3	23.1	3	13.6	6	17.1
Other		0	0.00	5	22.7	5	14.3
Total of cases		13	100	22	100	35	100
Total of respondents		7		15		22	

*Note:* This question allows multiple options. Therefore, the total number of responses can be higher than the number of participants.

The other respondents (*n* = 167) stated they used patients' feedback for many purposes. Thirty‐two percent of practitioners in this group reported that they used patient‐reported data for eliminating or limiting a critical aspect emerged by the patient voice, 55% for both solving negative aspects and valuing positive ones with a difference between the two Regions: namely, in Region B there is much more focus on the negative aspects (Table 3).

**TABLE 3:** Purposes of using patient‐reported data by Italian region and healthcare organisation.

Purposes	Region A		Region B		Total	
	Frequency	Percentage	Frequency	Percentage	Frequency	Percentage
To eliminate a critical aspect	14	15.7	40	51.3	54	32.3
To value a positive aspect	12	13.5	9	11.5	21	12.6
Both aspects	63	70.8	29	37.2	92	55.1
Total	89	100	78	100	167	100

By analysing the open‐ended questions, four themes (called ‘Areas for improvement’) emerged, namely, internal actions addressed to hospital staff (Theme A), external actions addressed to users (Theme B), comfort and hospitality aspects (Theme C) and review of processes and procedures (Theme D) (Table 4). The most mentioned uses of patient data are about the first and the third themes.

**TABLE 4:** Areas for improvement by Italian regional healthcare systems.

Areas for improvement	Region A		Region B		Total	
	Frequency	Percentage	Frequency	Percentage	Frequency	Percentage
Theme A: Internal actions addressed to hospital staff	35	39.3	25	32.1	60	35.9
Theme B. External actions addressed to users	16	18.0	15	19.2	31	18.6
Theme C. Comfort and hospitality aspects	30	33.7	28	35.9	58	34.7
Theme D. Review of process and procedures	8	9.0	10	12.8	18	10.8
Total of responses	89	100	78	100	167	100

#### Theme A: Internal actions addressed to hospital staff

The actions derived from patient feedback primarily focussing on initiatives targeting hospital staff included mostly internal meetings and training.

Internal meetings were conducted for reading, evaluating and learning from the patients' feedback, mostly qualitative one. These meetings aimed to understand the positive and negative aspects perceived by patients, thereby identifying areas for improvement in healthcare staff behaviours or care aspects.

Reading the comments of citizens were impactful.

(Region A, LHA1)

Where specified, these meetings were meant as structured periodic meetings, both purposively organised or already existing (i.e., standard doctor‐nurse meetings where a specific time frame was devoted to learning from patient‐reported data).

The patients' positive comments were used for motivating and valuing the staff. Professionals emphasised that patients reported, through PREMs survey, positive comments related to kindness of reception, clarity of information, emotional support and availability of staff. Notably, references to professionals' behaviours that have made a difference for patients during hospital stay were disseminated among the team, both individually and in groups, to reward professionals and reinforce the best practices.

Thanks to the positive narratives on the reception and treatment received by hospital staff, motivating actions to personnel were promoted.

(Region A, LHA1)

We found many positive comments for professionals. A poster has been hung in the wards where the nursing coordinators periodically insert some comments aimed at enhancing the staff […].

(Region B, TH2)

Moreover, structured training courses were planned. A threefold aim of these courses was found. A first kind of training actions was reported as aimed to increase the response rate to the PREMs survey. They were designed to respond to an internal analysis of the patients' participation in the PREMs survey, that pushed hospitals to focus the training on this aspect. During these training actions, professionals were provided with information and communication skills needed for patient engagement in the survey. This is because, in phase of enrolment, professionals could inform and encourage patients in participating. Second, training also represented the opportunity to disseminate data, so activating a virtuous circle of learning from patient‐reported data moving from dissemination of data to the uptake of them into new actions.

[…] encourage other professionals to use this tool.

(Region A, LHA1)

The third main purpose of the training actions was to improve the hospital performance on the patient experience. In this case, one of the key topics is related to kindness and quality of communication to and with patients and families. An example of training courses on specific topics was: ‘[…] training course on effective and non‐violent communication in neonatology to improve staff empathy with parents […]’. (Region B, LHA4).

#### Theme B: External actions addressed to users

Respondents have activated on‐field activities for facilitating effective communication between front‐line professionals and patients, especially when a negative performance on this experiential dimension was detected in the process of learning from patient‐reported data. In particular, some of these actions were designed in response to patients' complaints regarding unclear and non‐aligned responses from different professionals. Other hospitals realised or updated information brochure for patients, for improving clarity of information.

Users report lack of information related to discharge. We created a brochure that clearly illustrates the paths from discharge at home. We hope that this will have a positive impact on the transfer of information to caregivers regarding the discharge of patients. The delivery will be recorded in the medical electronic record.

(Region B, LHA6)

Attention has also been paid to information during discharge ‘to avoid misunderstanding and to increase the perceived quality’ (Region A, LHA1).

Actions have also been devoted to introducing structured communication practices for making clearer the doctor‐patient communication.

One patient complained that she was discharged after surgery without antibiotic therapy. Now, the doctors who deliver the discharge letter always specify that the post‐operative antibiotic therapy is not necessary because the intraoperative prophylaxis was performed. The goal is to prevent the patient thinks about a forgetfulness on our part and increase the perceived quality.

(Region A, LHA1)

Communicative and informative actions have also been targeted to caregivers, by investigating critical issues reported by family relatives in the PREMs survey. Caregivers mentioned lack of information during hospitalisation and concerns about the post‐discharge phase of care. Actions to improve communication were designed at least in two ways, namely: first, by providing more detailed information during hospital stay and at the discharge phase about health and social care support and services; second, by more clearly structuring the communication process, for example, identifying specific times for the professionals‐caregivers encounters.

[…] the doctor's communication with family relatives as a critical aspect. For this reason, we have regulated the communication of the surgeon both at the end of the surgery and during hospital stay in the ward.

(Region B, LHA4)

#### Theme C: Comfort and hospitality aspects

Several interventions for improving hospital comfort and physical environment were promoted by professionals. Many actions have been implemented to reduce noise, especially at night, such as awareness campaigns, changing visiting hours for family relatives or reorganising daily activities, as proposed by patients.

To improve the comfort of patients, caregivers, staff, have been activated behaviours aimed at reducing noise, especially at night. One of these consists in the posting of posters that remind that the ‘silence helps care and depends on everyone’. The action was implemented later reading the comments from which it emerged the discomfort of patients in being able to rest during the night. […] The PREMs is a powerful tool because it can answer people's needs during a delicate period of their lives.

(Region A, TH1)

Additionally, patient‐reported data were used to improve the cleanliness of departments, by sharing patients' comments to the external cleaning service providers, for stimulating opportunities for learning from patients and better control the suppliers' activity.

We found negative comments about cleanliness in the department. Comments are sent monthly to the Medical Department, Clinical Risk and the cleaning external service […] We expect that the action will allow us to take into account, as part of the ordinary control activity, the comments received through the PREMs survey and to detect any non‐compliance.

(Region B, TH2)

Other interventions addressed problems related to the food service, temperature, lighting and structural improvements to enhance patient comfort.

we opened a nutritional clinic, […] we involved new professional and expanded dietary counselling […].

(Region A, TH1)

The patients' feedback was also taken into account for restructuring and re‐design of the new wards.

Request to change the position of the nest with respect to the hospitalization area to prevent noise.

(Region B, LHA4)

The inpatient spaces have been modernised and adapted to patient requirements, by purchasing:

[…] new television sets.

(Region A, TH1)

[…] new seats (to be replaced with uncomfortable ones) for the rest of the parents of young patients.

(Region B, LHA6)

[…] new customized baby‐changing tables in some hospital rooms.

(Region B, LHA6)

#### Theme D: Review of Processes and Procedures

Professionals have taken proactive actions to reduce patients' criticisms about long waiting times from reception to surgery, by evaluating and reviewing existing administrative processes and procedures. The initiatives involved increasing acceptance points and evaluating different hospitalisation schedules for patients.

After the implementation of the action, we already note less congestion and better organization of the department […] the impact of the action will be monitor through patient experience survey.

(Region A, TH1)

[…] to monitor the flow of patients and caregivers in and out […] this action is also linked to the prevention of hospital infections.

(Region A, LHA2)

Additionally, some actions include the integration of new professionals into the team to optimise patient management process. The following example refers to the introduction of a nurse with specific roles and responsibilities related to comprehensive care and hospital‐primary care integration, called the pathway nurse (‘infermiere di percorso’).

In order to better organize the discharge phase and communication, a new professional figure has been introduced in the first half of 2022 in the medical department. It will become a reference figure for patients and caregivers in the preparation of the patient's discharge for a better taking care and re‐entry at home or at other healthcare services.

(Region B, LHA4)

Also, organisational models were adapted according to the patient‐reported data. Professionals from Region A, LHA3 mentioned the identification of dysfunctional models using PREMs feedback, and the introduction of a model of primary nursing in the hospital, with a referent nurse for patients and the development of individual care‐plans in the medical setting of the hospital.

Among the revisions of administrative procedures, adjustments to the nursing organisational model and reassessment of staff shifts were not only reported, but also disseminated.

Thank to the PREMs survey, the new organizational model appears to be better and more efficient than the previous one. For this reason, the new model was presented in other structures of the organization.

(Region B, LHA4)

#### 3.2.1 Impact and Evaluation

In around the 9% of cases (*n* = 15), the respondents explicitly reported to do not know what kind of monitoring, evaluation or control of the actions have or will been implemented. Sometimes the impact is reported as self‐evident. For example, the actions regarding the improvement of the communication were presented as an effective way to create a positive environment, so positively affecting the whole experience of patients, but also professionals.

Improvement of the perception of work quality and a good climate among patients, clinicians and nurses […].

(Region A, TH1)

These actions were also presented as cost‐effective.

It is not an effort and makes work better, making patients feel better.

(Region A, LHA1)

In the most of cases (*n* = 86; 51%), the practitioners explicitly reported the use of the same data from the PREMs Observatory for evaluating the effectiveness of the intervention, since it is an ongoing and permanent tool of data collection and reporting that allows for comparisons over time.

Additionally, other mechanisms of evaluation of the actions' impact have been reported, namely, the enhancements of clinical outcomes (e.g., reducing falls and reducing readmissions within 30 days) and improvements in performance results (e.g., indicators, standard, budget goals).

In the following example, the practitioners reported the expected impact, the goal to reach and the evaluation measure that can be daily monitored on the PREMs reporting system.

We expect an impact of the activity on the care relationship between operators and parents/newborn. The goal is to improve parents' assessment of emotional support, communication and involvement by 15‐20%. they must feel fairly supported and understood by the staff.

(Region B, LHA4)

#### 3.2.2 Determining the Validity of the Data

Results from the second phase were validated through a final workshop. The workshop was much more participated compared to the first, with 43 participants attending in person and approximately 150 joining remotely. This underlines the increased interest among practitioners. This final workshop was attended by the coordinators of the PREMs observatory with managerial role, as well as front‐line professionals.

Participants expressed their willingness to learn from the others' experiences. Several questions were proposed to better understand how the other professionals used the patient‐reported data and implemented the actions, in order to understand the feasibility of adopting the same practices. Participants discussed actions at the different levels of the organisation (micro, meso and macro). At the macro levels, questions about indicators of evaluation, goals and standards were mostly discussed. At the meso level, professionals gave more attention to actions regarding the promotion of the silence and the evaluation of cleaning services. At the micro level, there was a great variety of activities discussed and taken as examples of future opportunities of patient‐reported data use by participants. They found the results of the survey very interesting and approved the interpretation of results presented. Most of all, the community of practice built around the PREMs Observatory was recognised as a powerful mechanism to share and increase knowledge, to learn and to promote the creation of common knowledge within the community itself, to be then disseminated within the organisations of origin.

## 4 Discussion

This study investigates how two slightly different approaches of patient data management initiate a virtuous cycle of knowledge dissemination, exchange and use. Specifically, the research focussed on whether and how patient‐reported experience data have been used in two settings, namely, two Italian regional healthcare systems.

Overall, the majority of respondents declared to have used patient‐reported data. This underlines the commitment of professionals on collecting patients' feedback, but especially on using patient‐reported data to improve healthcare services. Moreover, the establishment of a PREMs collaborative network, functioning as a community of practice, seems to have reduced the learning curve and experience in using patient‐reported data. It increases familiarity and experience with patient‐reported measures accelerating process over time. It may have encouraged healthcare organisations to use the data through comparison, mutual inspiration and experiential learning, shifting the emphasis from mere data collection to effective use of patient voice. From the analysis, there seems to be a path dependency and a reduction in the learning curve, as well as a kind of positive contagion and passion in the use of this data [49]. This finding can also suggest that patient feedback has becoming a common knowledge for healthcare organisations. The dissemination of patient data across the organisations makes it easily to believe that the possessors of this knowledge are not only managers anymore, but all the professionals working into the organisation. It can be stated that the presence of learning‐systems is observed, as indicated by McHugh, Groves, and Alker [19]. According to their words, genuine learning organisations entails individuals within the process driving organisational transformation. In this context, individuals are the engines of the transformative actions, rather than the organisation using learning as a tool to shape and influence individuals. The choice of sharing at all levels of the organisation the patient experience data, the workshops directly involving practitioners and the actual use of this knowledge show that there is an ongoing process of blending some features of a learning organisation, such as empowerment, trust and communication [50]. This shift from mere data benchmarking to practices benchmarking may facilitate the transition of systems towards becoming true learning organisations.

The results reveal a difference between the two regions, with a higher percentage of practitioners reporting extensive use of this kind of information in Region A. This can also be partially attributed to path dependency: Region A began its involvement with the PREMs Observatory in a more systematic and widespread manner, building also upon a long‐standing history of patient data collection that existed prior to the PREMs Observatory establishment. Another explanation can be found in the fact that the patient‐information was integrated into the PES as indicators, suggesting that the combination of full data‐availability, collaborative workshops, and harder managerial mechanisms can make the system more oriented towards learning from patients [50]. In fact, the two regions have had slightly different approaches to promoting the consideration and use of knowledge from the PREMs Observatory. In both regions, on the one hand the dissemination of data was capillary and prompt, and on the other hand the PREMs collaborative network promoted the sharing of practices, but only one region (Region A) adopted also patient indicators integrated into its PES.

The findings report a notable concentration on activities aimed at internal staff, such as internal meetings and training actions. This aligns with previous studies that highlight an association between training, multidisciplinary group meetings and sharing of practices with a better patient experience [39]. In addition, using patients' positive feedback to motivate professionals and involve them into quality improvement actions could lead to better care and better patient experience. Training courses for hospital staff were planned to increase the knowledge of the tool and awareness of the collection and use of the patients' feedback. As demonstrated by Murante et al., disseminating data within organisations has an impact in driving behavioural changes in professionals [5]. Training or motivational activities targeted to the internal staff are usually implemented at the hospital or healthcare organisation level. Thus, this result can be explained by the fact that the integration of patient indicators in the PES in Region A affects mainly managers of the healthcare organisations and hospitals, then falling back on professionals at the ward levels. This process could have been pushed by these mechanisms of management control, so incentivizing managers and practitioners to promote actions informed by this kind of knowledge.

The second most common use of patient data was to improve hospital comfort and physical environment, which practitioners considered small changes that do not require a change in clinician behaviour [26]. This does not apply to noise when coming from chatting of professionals or during the shift change in the ward, which has been reported among the actions in this study. Future studies could focus on organisational efforts to change the professionals' behaviours affecting the comfort.

Finally, contrary to prior studies where the most common changes related to administrative procedures (e.g., appointment scheduling for decreasing waiting time) [23, 26], in this study such initiatives were the least frequent. Despite this, reviewing procedures for reducing waiting times is a crucial practice, also leading to improved experience of patients and hospital staff [51].

This study identifying and mapping actions based on patient information, with a knowledge‐based approach, has also managerial implications. First, managers maximising the utilisation of knowledge to achieve the organisational mission, strategies, and goals should incentivize the access to explicit and understandable information, as the PREMs Observatory does, but also to the tacit knowledge. Extraction, documentation, sharing, and utilisation of this tacit knowledge could be facilitated by a process of organisation‐level sharing (i.e., mapping of actions, sharing of best practices). This is additionally important considering that a deep knowledge of initiatives of quality improvement can facilitate a more efficient use of resources [52]. This dissemination of knowledge to front‐line staff, practitioners and managers cultivates a culture of continuous learning and improvement [52]. Second, combining different managerial levers of knowledge dissemination and use is fundamental. It seems that the capillary dissemination of knowledge is essential [5], but not sufficient alone to making the patient data a common knowledge‐base for improving the quality of services. The PREMs Network, functioning as community of practice, play a crucial role in supporting patient data use. In the community of practice, peer learning, common support and shared experiences help to translate knowledge into quality improvement practices. Moreover, traditional managerial mechanisms of monitoring, control, evaluation and incentive appear still determinant in shaping organisational culture and incentivizing the use of patient‐reported data [10]. The integration of different knowledge management strategies seems to support the establishment and enhancement of learning systems, where data benchmarking is combined with benchmarking of practices, and capillary knowledge dissemination as well. These mechanisms may activate both top‐down and bottom‐up dynamics, driving the transition towards more effective, patient‐centred healthcare practices.

## 5 Limitations and Directions for Future Research

This study is not free from limitations.

Firstly, the study is based on the self‐reported practices from practitioners regarding their use of patient‐reported data. This may introduce a self‐selection bias, as those more engaged with patient data could be overrepresented in the study. Further research could investigate not only the use of patient‐reported data, but also its impact on patients, caregivers, professionals and organisation.

Secondly, the research proposes a snapshot of improvement actions and initiatives based on patient‐information implemented in two Italian regional healthcare systems. However, learning processes could evolve over time, potentially influencing other professionals and organisations in making use of this knowledge. Further studies could conduct longitudinal analysis for exploring determinants, time and efforts needed to shift from data collection and data use, by considering the presence (or not) of a collaborative community of practice.

Thirdly, the practices reported by practitioners may have been influenced by a top‐down approach. Future exploration could consider the role of top management on the use of patient‐reported data and map actions voluntarily implemented by professionals.

## 6 Conclusions

This research examines how patient‐reported data can serve as a knowledge base for improving healthcare services and patient experience.

The findings reveal slight significant differences between the two Italian regional healthcare systems, in terms of the extent and focus of actions derived from patient‐experience data. In particular, the region that integrate patient indicators into the performance evaluation system demonstrated a wider use of knowledge from patient‐reported information into quality improvement actions. Despite slight variations emerged in the target of the actions, both regions primarily target internal aspects, such as staff training and comfort improvements, while also addressing communication issues with patients and caregivers.

This research highlights the pivotal role of knowledge dissemination in driving improvement actions, advocating for a culture of continuous learning and enhancement within healthcare organisations. Additionally, the presence of different reporting systems underscores the need for tailored strategies to effectively use patient‐reported data across diverse healthcare settings, with typical managerial mechanisms of performance management and control playing a positive role.

In conclusion, the paper emphasises the transition from merely comparing patient‐reported data to benchmark practices. Establishing learning systems, with a combination of knowledge management approaches, can optimise the utilisation of patient‐reported experience measures, ultimately aiding the progression towards more efficient healthcare practices.

Overall, this study contributes to the ongoing discourse on patient‐centred care and underscores the importance of harnessing organisational processes of learning to drive meaningful improvements in healthcare delivery.

## Conflicts of Interest

The authors declare no conflicts of interest.

## Supporting information

Supplementary Material S1

Table S1

## Data Availability

Data are available from the corresponding author on reasonable request.
