# Supplementary material for: Enhancing Learning Systems in Using Patient Experience Data: An Exploratory Mixed‐Method Study in Two Italian Regions
Source: Int J Health Plann Manage. 2025 Feb 25;40(3):688–700. doi: 10.1002/hpm.3912 (PMC12045758; doi:10.1002/hpm.3912)

**Supplementary Materials**

**FIGURE 1** Patient-reported data represented through web-based platform


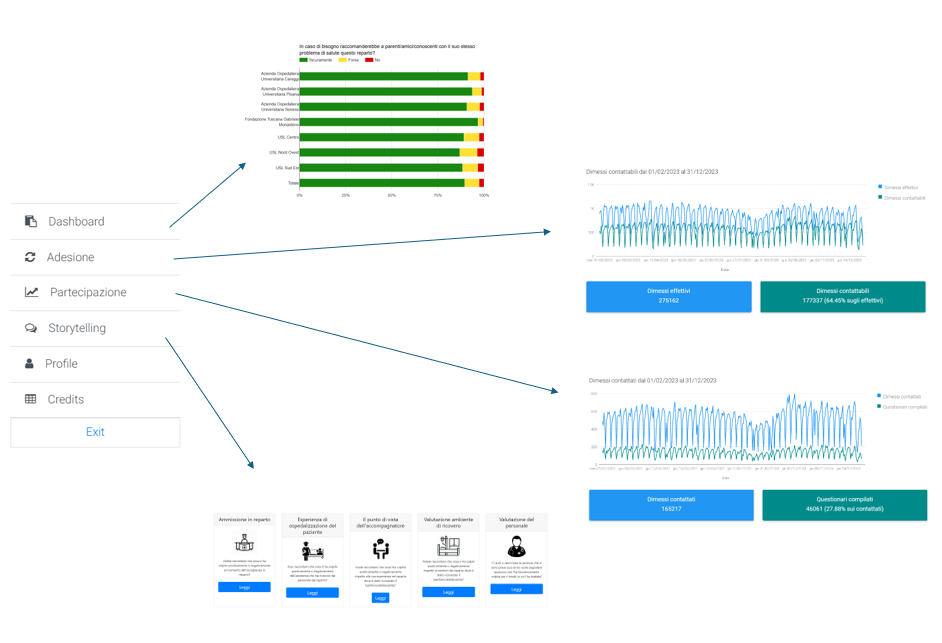


**FIGURE 2**  Indicator from the PREMs Observatory - Health authorities, hospital and trend histogram


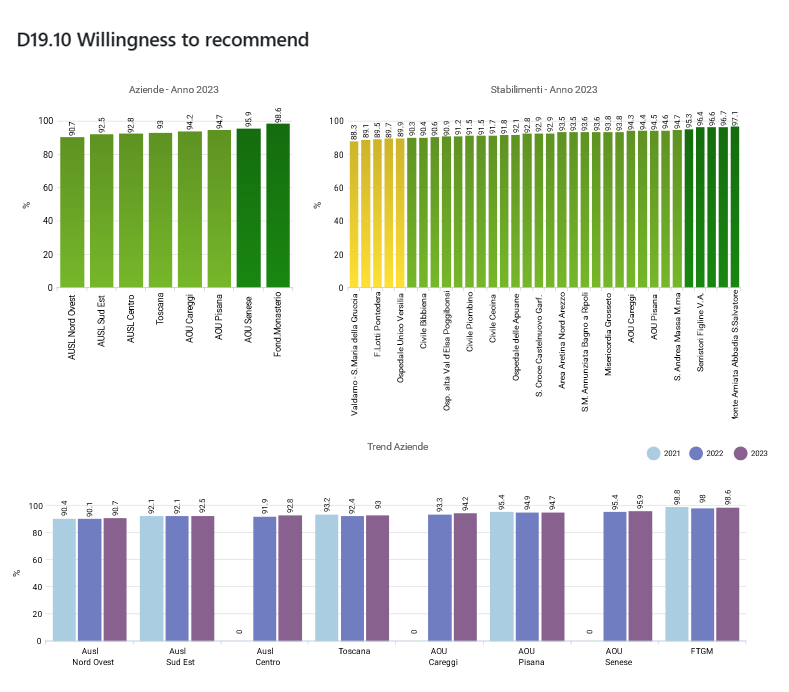

Supplement: Supplementary file 1 — Supplementary Material S1 [file HPM-40-688-s002.docx]
