# Supplementary material for: Enhancing Learning Systems in Using Patient Experience Data: An Exploratory Mixed‐Method Study in Two Italian Regions
Source: Int J Health Plann Manage. 2025 Feb 25;40(3):688–700. doi: 10.1002/hpm.3912 (PMC12045758; doi:10.1002/hpm.3912)
Supplement: Supplementary file 2 — Table S1 [file HPM-40-688-s001.docx]

**Supplementary material**

**TABLE 1** Regions, organizations and hospitals involved in the study.

| **Region** | **Organization** (type, n°) | **Hospitals (n°)** |
| --- | --- | --- |
| Region A | LHA1 | 9 |
|  | LHA2 | 9 |
|  | LHA3 | 11 |
|  | TH1 | 1 |
| ***Total Region A*** | ***4*** | ***30*** |
| Region B | LHA4 | 6 |
|  | LHA5 | 5 |
|  | LHA6 | 5 |
|  | TH2 | 2 |
| ***Total Region B*** | ***4*** | ***18*** |
| ***Total in the two Regions*** | ***8*** | ***48*** |
